# Mucosal melanoma of the head and neck: a population-based study from Slovenia, 1985-2013

**DOI:** 10.1186/s13014-016-0712-9

**Published:** 2016-10-14

**Authors:** Gaber Plavc, Jasna But-Hadžić, Aleksandar Aničin, Boštjan Lanišnik, Vojislav Didanović, Primož Strojan

**Affiliations:** 1 Department of Radiation Oncology, Institute of Oncology, Ljubljana, Slovenia; 2 University Department of Otorhinolaryngology and Cervicofacial Surgery, University Clinical Center, Ljubljana, Slovenia; 3 Department of Otorhinolaryngology, Cervical and Maxillofacial Surgery, University Clinical Center, Maribor, Slovenia; 4 Department of Maxillofacial and Oral Surgery, University Clinical Center, Ljubljana, Slovenia; 5 Faculty of Medicine, University of Ljubljana, Ljubljana, Slovenia

**Keywords:** Mucosal melanoma, Head and neck cancer, Epidemiology, Therapy, Survival

## Abstract

**Objectives:**

To assess the incidence and to review experience with the treatment of mucosal melanoma of the head and neck (MMHN) in Slovenia between 1985 and 2013.

**Methods:**

The National Cancer Registry database and clinical records with outcome data of identified patients treated during the period 1985–2013 in Slovenia were reviewed.

**Results:**

In a 29-year period, 61 patients with MMHN were identified, representing 0.5 % of all head and neck malignant tumors and 42 % of all mucosal melanomas in Slovenia. 72 % originated in the sinonasal tract and were predominantly (78 %) diagnosed as a local disease. Regional metastases at diagnosis were more frequent in patients with oral/oropharyngeal primary (44 %; sinonasal MMHN 11 %, *p* = 0.006). Curative intent treatment was given to 48 (79 %) patients. The overall survival (OS) rates at 2 and 5 years for the whole cohort were 43 % and 18 %, respectively, and for the curative intent group 53 % and 24 %, respectively. In the latter group, multivariate analyses showed postoperative radiotherapy (PORT) to be predictive for locoregional control (LRC) (hazard ratios [HR] for surgery with PORT vs. surgery alone: 1.0 vs. 3.9, *p* = 0.037), whereas only the World Health Organization performance status (HR for grade 0 vs. grade 1 vs. grade >1: 1.0 (*p* = 0.022) vs. 1.2 (*p* = 0.640) vs. 7.7 (*p* = 0.008)) significantly influenced OS.

**Conclusions:**

MMHN is a rare tumor with a poor prognosis. Combination of surgery and PORT offers the best prospects for LRC but without improvement of OS. Due to potential toxicity of high-dose RT such treatment is indicated in patients in whom LRC outweighs the risks of serious adverse effects.

## Introduction

Mucosal melanoma of the head and neck region (MMHN) was first described by Weber in 1856 and has since been the subject of an increasing number of retrospective studies while its rareness precluded any prospective trials [1]. The yearly incidence of mucosal melanoma is 2.2–2.6 cases per million and approximately half of the cases appear in the upper part of the aerodigestive tract [2–4]. Higher MMHN incidence was reported in Japan and Uganda, although the ratio of MMHN vs. all mucosal melanomas seems to be the same for Caucasians and the Japanese [5, 6].

Malignant melanomas derive from melanocytes or their precursor cells arising from the neural crest [7]. Contrary to its cutaneous counterpart, strong evidence of environmental risk factors for the development of mucosal melanoma is lacking and the possible role of premalignant lesions remains to be elucidated as well [8]. Diagnosis of pigmented MMHN is usually unambiguous, however, it can be difficult to obtain in amelanotic and ulcerated lesions [9]. Regardless of treatment, overall survival (OS) in MMHN is poor and rarely do 5-year OS rates exceed 30 % [8]. It is generally agreed upon that radical surgical resection of the primary tumor offers the best chance of local control and cure, whereas the role of elective neck resection and adjuvant-postoperative radiotherapy (PORT) is not well established [8].

In the present study we sought to describe the incidence of MMHN in Slovenia for the period from 1985 to 2013, to report our experience with these patients, and to assess the significance of previously proposed prognostic factors.

## Patients and methods

### Patient and tumor characteristics

Patients diagnosed from 1985 through 2013 with MMHN in Slovenia were eligible for this nationwide population-based retrospective study. In total, 61 consecutive patients were identified by the Cancer Registry database, a population-based cancer registry covering the entire Slovenian population since 1950 [10].

Epidemiological and clinical parameters are presented in Table 1. Tumors were most frequently located in the sinonasal tract (SN) (44 patients, 72 %) with subsites of origin as follows: nasal cavity (33 patients, 54 %), maxillary sinus (6 patients, 9.8 %), ethmoid sinus (3 patients, 4.9 %) and nasopharynx (2 patients, 3.3 %). Sixteen tumors (26 %) originated from the oral cavity (14 patients, 23 %) and oropharynx (2 patients, 3.3 %) (OC-OP). One patient presented with MMHN of the middle ear.

**Table 1:** Epidemiological and clinical parameters of 61 patients with MMHN diagnosed in Slovenia, 1985–2013

Characteristic	All cases	Sinonasal tract	Oral cavity & oropharynx	*p*-value
	*N* = 61 (%)	*N* = 44 (%)	*N* = 16 (%)	
Age at diagnosis (years)				
Median	75.5	77.0	68.2	0.059
Range	25.9–91.8	40.0–91.8	25.9–87.3	
Sex				
Male	32 (53)	20 (46)	11 (69)	0.110
Female	29 (48)	24 (55)	5 (31)	
PS WHO				
Grade 0	29 (48)	18 (41)	10 (63)	0.276
Grade 1	23 (38)	18 (41)	5 (31)	
Grade >1	9 (15)	8 (18)	1 (6.3)	
Duration of symptoms (months)				
Median	2.0	2.0	1.0	0.554
Range	0.0–12.0	0.0–12.0	0.0–12.0	
Tumor pigmentation				
Yes	36 (59)	23 (52)	13 (81)	0.165
No	13 (21)	11 (25)	2 (13)	
Unknown	12 (20)	10 (237)	1 (6.3)	
Overall stage				
1	48 (79)	38 (86)	9 (56)	0.029
2	10 (16)	4 (9.1)	6 (38)	
3	3 (4.9)	2 (4.5)	1 (6.3)	
TNM stage				
III	21 (34)	16 (36)	4 (25)	0.203
IVA	30 (49)	19 (43)	11 (69)	
IVB	7 (12)	7 (16)	0 (0.0)	
IVC	3 (4.9)	2 (4.5)	1 (6.3)	
Treatment intent				
Curative	48 (79)	33 (75)	14 (88)	0.299
Palliative	13 (21)	11 (25)	2 (13)	
Surgery				
No	22 (36)	20 (46)	2 (13)	0.019
Yes	39 (64)	24 (55)	14 (88)	
Open surgery	24 (62)	10 (42)	13 (93)	0.002
Endoscopic surgery	15 (39)	14 (58)	1 (7.1)	
Radiotherapy				
No	28 (46)	21 (48)	7 (44)	0.785
Yes	33 (54)	23 (52)	9 (56)	
Definitive	11 (33)	9 (39)	2 (22)	0.066
Postoperative	16 (49)	8 (35)	7 (78)	
Palliative	6 (18)	6 (26)	0 (0.0)	
Systemic therapy				
Yes	13 (21)	10 (23)	3 (19)	0.741
No	48 (79)	34 (77)	13 (81)	
2-year overall survival^a^	43 (30–55)	40 (26–55)	54 (29–79)	0.548
5-year overall survival^a^	18 (7.8–29)	20 (7.4–32)	15 (0.0–35)	0.548

^a^% (95 % confidence interval). *N* Number of patients

At presentation, localized disease was found in 48 patients (789 %) with 21 (44 %) tumors staged as T3, 22 (46 %) as T4a and 5 (10 %) as T4b (UICC TNM, 7th ed.). Twelve patients (20 %) presented with positive cervical lymph nodes (CLN) and three patients (4.9 %) were diagnosed with distant metastases: one of these patients had lung metastases, while the other two presented with metastatic spread to multiple sites (Table 1). Presenting symptoms and initial diagnostic work-up are summarized in Table 2.

**Table 2:** Presenting symptoms and initial diagnostic work-up

Presenting symptoms	No. of patients (%)
Epistaxis	20 (33)
Evident mass or swelling	18 (30)
Nasal obstruction	17 (28)
Pain	3 (4.9)
Regional non-nasal bleeding	2 (3.3)
Hearing impairment	2 (3.3)
Headache	1 (1.6)
Dysphagia	1 (1.6)
Signs of metastatic disease	1 (1.6)
No symptoms reported	17 (28)
Duration of symptoms (months)	
Range	0.0–12.0
Median	2.0
Diagnostic work-up	
Local tumor extension	
CT	31 (51)
Endoscopy	19 (31)
MRI	5 (8.2)
Sinus X-ray	5 (8.2)
Clinical examination only	16 (26)
Regional node involvement	
US	31 (51)
CT	2 (3.3)
MRI	1 (1.6)
Clinical examination only	29 (48)
Distant metastatic spread	
Chest X-ray	45 (74)
Abdominal US	31 (51)
Bone scan	4 (6.6)
Thoracic CT	3 (4.9)
PET-CT	3 (4.9)
Head CT	1 (1.6)
Clinical examination only	12 (20)

*CT* Computer tomography, *MRI* Magnetic resonance imaging, *US* Ultrasonography, *PET* Positron emission tomography

### Treatment

Forty-eight patients (79 %) were treated with curative intent, nine (14.8 %) received palliative treatment and four (6.6 %) had only symptomatic treatment.

#### Treatment of primary tumor

In the curative intent group, the first-line therapy was surgery in 37 patients (77 %) and definitive RT in 11 (23 %). There was no statistically significant difference either in TNM stage distribution or in World Health Organization performance status (WHO PS) between these two groups. Surgery was either open (22 patients, 59 %) or endoscopic (15 patients, 41 %) with clear margins achieved in 31 cases (84 %). PORT was delivered in 16 (43 %) patients, from 20 to 65 days after surgery (median, 45 days) (Table 1). In five cases the indications for adjuvant RT to the primary tumor bed were positive margins of resection, while in the other 11 cases the decision for adjuvant RT was based on SN localization (7 cases), high local disease burden (pT4, seven patients) or regional spread (pN+, three patients). Six out of 11 primarily irradiated patients had a complete response locally (assessed 8 to 12 weeks after RT by local clinical examination only), four had a partial response and one patient was declared a non-responder. No patient was surgically salvaged, the reasons being an inoperable disease or the patient's general condition being deemed unsuitable for surgery. In the PORT and definitive RT groups the equivalent RT doses to the primary tumor site in 2-Gy fractions (EQD_2,_ α/β = 2 Gy) [11] ranged from 45.0 to 72.0 Gy (median, 60.0 Gy) and from 52.5 to 75.0 Gy (median, 68.8 Gy), respectively. RT was delivered using conventional fractionation of five 2.0–3.0 Gy fractions (median, 2.0 Gy) per week in 17 cases, and hypofractionation of 2–3 weekly fractions of 4.0–6.0 Gy (median, 6.0 Gy) in 10 cases.

#### Treatment of neck

Eight patients had neck dissection, of whom six had clinically palpable CLN; metastatic nodes were found in all six patients. Four of these patients had PORT (45.0–66.0 Gy, median 65.0 Gy) due to pN+ disease (three patients, two with extracapsular tumor spread) and synchronous squamous cell carcinoma of the tongue base spreading to the CLN (1 patient). In patients without neck surgery, CLN were irradiated in six cases, four of them having had bilateral RT (63.0–72.0 Gy, median 70.0 Gy). Two of these patients had clinically positive CLN; complete and partial response was clinically recorded after RT in one patient each.

#### Radiotherapy technique

Two-dimensional computer planning, three-dimensional conformal technique (3D-CRT) and intensity-modulated technique (IMRT) were employed in 15 (56 %), eight (30 %) and three (11 %) patients, respectively, whereas in one patient RT was delivered by direct opposing field (3.7 %). RT was delivered by a 12 MeV electron beam, megavoltage cobalt-60 and 5–10 MV photon beams in one, six and 20 patients, respectively. After the year 2007, 3D-CRT and IMRT were introduced (and cobalt-60 irradiation was abandoned) in all the patients receiving radiotherapy.

#### Adjuvant systemic therapy

It was administered to 10 patients treated with curative intent (21 %). In 6/10 low-dose interferon α-2b (3 million IU i.m., three times/week for 25–60 weeks) was given after the primary operation, two patients had chemotherapy (vinblastine-lomustine-cisplatin, three cycles; dacarbazine, nine cycles) after RT and two patients were treated with concurrent PORT and low-dose interferon α-2b.

#### Palliative treatment

It was offered to nine patients and consisted of either local excision or debulking of the primary tumor (2 patients), palliative RT (6 patients, EQD_2_ 24–68 Gy, median 53.6 Gy) or systemic therapy only (1 patient).

### Statistical analyses

The study protocol was approved by the Protocol Review Board of the Institute of Oncology Ljubljana (ERID-KESOPKR/47).

The survival times were calculated from the start of treatment and were censored at the close-out date (February 15th 2015). The end points considered were local control (LC), regional control (RC), locoregional control (LRC), distant metastasis-free survival (DMFS), and OS (death from any cause considered as an event). The Kaplan-Meier method was used for univariate analysis of survival estimates and the differences between potential prognostic groups were tested by a log-rank test with 95 % confidence intervals (CI) reported. The hazard ratio (HR) calculations and multivariate analysis of significant prognostic factors from univariate analysis were performed by the Cox proportional hazard regression model. The number of covariates to be included in the multivariate analysis was determined according to Peduzzi et al. recommending 10 events per variable [12]. All statistical tests were 2-sided and a *p*-value < 0.05 was considered significant.

## Results

### Epidemiology

In the 29-year period from 1985 to 2013, MMHN represented 0.5 % of all head and neck malignancies, 0.8 % of all melanomas, 4.4 % of all melanomas in the head and neck region and 42 % of all mucosal melanomas in Slovenia.

### Follow-up

The length of the follow-up period for all 61 patients ranged from 0.9 to 190.2 months (median, 16.5 months), and 22.5 months (range, 0.9–190.2 months) and 8.8 months (range, 1.6–25.7 months) for those treated with curative and palliative intent, respectively.

### Patterns of failure

Out of 48 patients treated with curative intent, 15 (31 %) patients eventually failed locally. The median time to local recurrence was 15.4 months (range, 3.4–69.2 months) and to regional recurrence, diagnosed in eight patients (17 %; 5/8 patients had no treatment to the neck) 7.7 months (range, 3.4–106.3 months) after the start of the treatment.

The 2- and 5-year LRC rates were 52 % (95 % CI 36–69) and 27 % (95 % CI 7.6–46), respectively. Within the group of operated patients the improvement in LRC with the addition of PORT was significant (*p* = 0.019) with LRC at 2 years in operated-only patients and those with PORT being 43 % (95 % CI 18–68) and 84 % (95 % CI 64–100), respectively, and at 5 years 18 % (95 % CI 0–40) and 67 % (95 % CI 33–100), respectively.

Systemic disease eventually developed in 24 (50 %) patients 0.9 to 145.5 months after therapy (median, 11.8 months; 95 % CI 4.2–23). The DMFS rates at 2 and 5 years were 58 % (95 % CI 42–73) and 40 % (95 % CI 22–57).

**Fig. 1:**
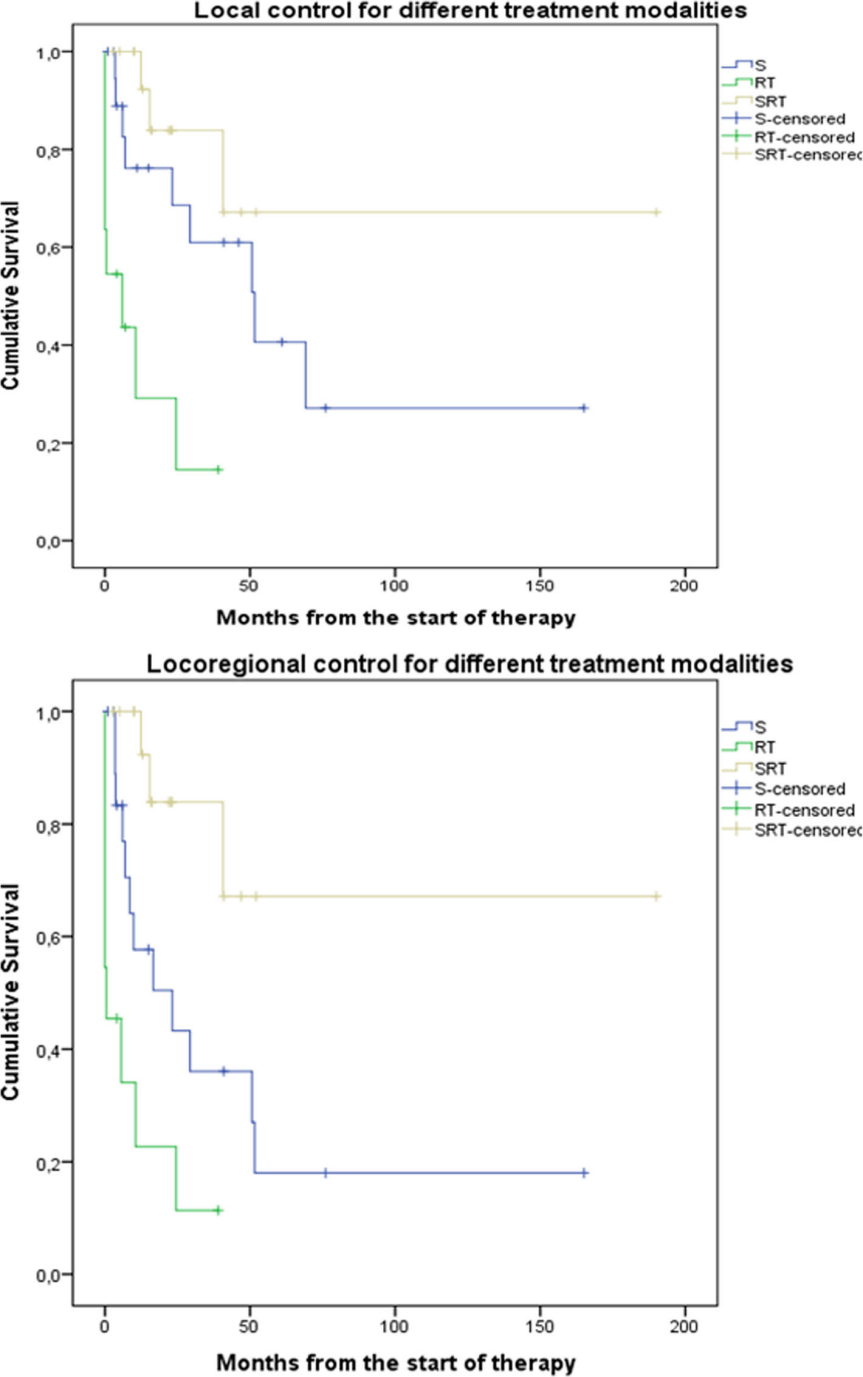
Impact of treatment modalities on local and locoregional control. S – surgery, RT – radiotherapy, SRT – surgery and postoperative radiotherapy

Several prognostic factors were confirmed to be statistically significant on univariate analysis in terms of influencing the LC, LRC, and DMFS (Table 3). In multivariate analysis, only treatment modality appeared significant for predicting LC (Fig. 1, Table 4).

**Table 3:** Univariate analysis

Prognostic factor	Outcome (*p*-value )*				
	LC	LRC	DMFS	DSS	OS
Age (years)					
< 65 vs. >65	0.188	0.135	0.083	0.074	0.036
Sex					
Male vs. Female	0.674	0.241	0.425	0.276	0.189
Performance status (WHO)					
0 vs. 1 vs. 2 + 3 + 4	0.034	0.095	0.860	<0.001	<0.001
Site					
SN vs. OC-OP	0.125	0.056	0.291	0.581	0.768
Pigmented lesion					
Yes vs. No	0.888	0.953	0.157	0.329	0.246
T-stage					
T3 vs. T4A/B	0.752	0.931	0.643	0.291	0.226
N-stage					
N0 vs. N+	0.612	0.650	0.892	0.741	0.742
TNM-stage					
III vs. IVA/B	0.595	0.708	0.807	0.382	0.315
Year of first treatment					
1985–2007 vs. 2008–2013	0.926	0.286	0.860	0.777	0.916
Treatment modality					
Surgery vs. RT vs. surgery + PORT	<0.001	<0.001	0.796	0.111	0.074
Systemic therapy					
Yes vs. No	0.858	0.376	0.036	0.022	0.011
Failure of local control					
Yes vs. No	NA	NA	0.994	0.802	0.905
Failure of regional control					
Yes vs. No	0.720	NA	0.498	0.636	0.472
Failure of distant control					
Yes vs. No	0.741	0.434	NA	0.088	0.234

*Regional control was not used in Kaplan-Meier calculations due to low number of events (*N* = 8).

*LC* Local control, *RC* Regional control, *LRC* Locoregional control, *DMFS* Distant metastasis-free survival; *DSS* Disease-specific survival, *OS* Overall survival, *WHO* World Health Organization, *SN* Sinonasal, *OC-OP* Oral cavity/oropharynx, *RT* Radiotherapy, *PORT* Postoperative radiotherapy, *NA* Not applicable

**Table 4:** Multivariate analysis

Prognostic factor^a^	LC		LRC		DMFS		OS	
	HR (95 % CI)	*p*-value	HR (95 % CI)	*p*-value	HR (95 % CI)	*p*-value	HR (95 % CI)	*p*-value
Age (years)								
<65	NI		NI		1.0		1.0	
>65					1.2 (0.40–3.4)	0.793	0.89 (0.31–2.6)	0.836
Performance status (WHO)								
0	1.0	0.523	1.0	0.473	NI		1.0	0.022
1	0.73 (0.24–2.2)	0.576	0.61 (0.23–1.6)	0.315			1.2 (0.52–2.9)	0.640
2+3+4	1.9 (0.31–12)	0.483	1.4 (0.25–7.6)	0.722			7.7 (1.7–34)	0.008
Site								
SN	2.1 (0.52–8.7)	0.298	2.1 (0.64–7.4)	0.216	NI		NI	
OC-OP	1.0		1.0					
Pigmented lesion								
Yes	NI		NI		1.0		NI	
No					1.2 (0.40–3.4)	0.779		
Treatment modality								
Surgery	2.2 (0.42–12)	0.355	4.7 (0.98–22)	0.054	NI		1.0 (0.46–2.2)	0.979
RT	11 (2.1–63)	0.005	16 (3.0–80)	0.001			2.0 (0.73–5.5)	0.175
surgery+PORT	1.0	0.005	1.0	0.002			1.0	0.303
Systemic therapy								
Yes	NI		NI		1.0		1.0	
No					2.6 (0.62–11)	0.193	3.1 (0.92–10)	0.068

^a^Factors with no statistical significance in univariate analysis were included if deemed clinically relevant

*NI* not included in multivariate analysis, *LC* Local control, *LRC* Locoregional control, *DMFS* Distant metastasis-free survival, *OS* Overall survival, *WHO* World Health Organization, *SN* Sinonasal, *OC-OP* Oral cavity/oropharynx, *RT* Radiotherapy, *PORT* Postoperative radiotherapy

### Survival and prognosis

In curative intent treatment group, salvage therapy was offered to 23 out of 30 patients (77 %) with recurrent disease, of whom nine were treated more than once. At the close-out date, 38 patients (79 %) had died of MMHN, five (10 %) were alive and free of MMHN, and four had died of other causes (with persistent MMHN occurring in one patient). For one patient the vital status was unknown.

The OS ranged from 0.9 to 190.2 months (median, 25.4 months; 95 % CI 2.5–48). The OS rates at 2 and 5 years were 53 % (95 % CI 39–67) and 24 % (95 % CI 11–36), respectively. Univariate analysis showed a significant impact of age (*p* = 0.036), PS WHO (*p* < 0.001) and systemic therapy (*p* = 0.011) on OS (Table 3). Besides these, treatment modality as a clinically relevant factor with marginal significance (*p* = 0.074) in univariate analysis was also introduced into the Cox multivariate model. However, only PS WHO was retained in the model (Table 4).

For the whole cohort of 61 patients, the OS rates at 2 and 5 years were 43 % (95 % CI 30–55) and 18 % (95 % CI 7.8–29), respectively.

### Long-term survivors

Nine out of 48 patients (19 %; six females and three males, from 28.6–77.8 years of age, median 75.5 years) survived for 5 years or more after being diagnosed with MMHN, of whom three lived 12.1, 13.8 and 15.9 years after diagnosis. The origin of primary tumors was the nasal cavity in seven cases and oral cavity in two cases. Primary tumor stage at diagnosis was T3 in five and T4a in four cases; one patient was presented with regional metastases. All patients had up-front surgery (endoscopic – 6, open – 3) with clear margins achieved in all cases. One patient had unilateral elective dissection of regions I-II and in another patient bilateral dissection was done due to clinically evident CLN metastases (regions I-V). PORT was implemented in two cases. Disease recurred in six patients: two of them had isolated local recurrence 41 and 51 months after the first treatment and were successfully salvaged by additional surgery with PORT in one case. The other four patients had more than one failure. Six of the long-term survivors eventually died of MMHN.

## Discussion

In the present study, surgery followed by PORT resulted in the most favorable LRC compared to surgery alone or RT alone. In view of no survival advantage of combined therapy observed in our patients and significant lack in the use of comprehensive pre- and post-RT imaging (CT/MR), we recommend PORT only to selected patients at increased risk of recurrence in surgical field.

The incidence of MMHN in Slovenia (1.1 per million per year) is in line with the published data from Europe and the USA and does not appear to be rising [2–4, 10]. Also, other epidemiological data are within the frames of comparable preceding reports [13–16]. MMHN's predilection to occur in the nasal cavity and paranasal sinuses compared to the oral cavity and oropharynx was confirmed in the present series, yet no explanation of this difference has thus far been validated [8]. In the present series, the mode of primary treatment did indeed affect the course of the disease. The most favorable outcome in terms of LC and LRC was achieved by combining surgery and RT (Fig. 1, Table 4), although with no survival advantage observed. Therefore, potential toxicity of high-dose RT should always be taken into account and such treatment offered only to selected patients where LRC outweighs the risks of serious adverse effects.

The efficacy of RT in MMHN has been thus far analyzed in a number of series and it seems that both definitive RT and PORT improve local control [17]. In particular, high-linear energy transfer radiation appears to be comparable to surgery in maintaining local control in MMHN [18]. Both positive and close margins, difficult-to-access SN sites with a high probability of residual disease as well as multiple positive nodes and extracapsular tumor spread warrant adjuvant irradiation [17]. In our patients photon/electron RT to the site of the primary tumor was employed in 27 (56 %) curative-intent patients with the neck irradiated in 10 of these patients to the median EQD2 of 68.8 Gy (definitive setting) and 60.0 Gy (PORT setting). The herein confirmed positive impact of high dose PORT (>54 Gy) on LRC has been previously suggested by Moreno et al. [19] In regard to fractionation pattern, the optimal schedule is unclear: conventional fractionation is widely accepted (it was used in 18/27 of our patients) whereas clinical utility of hypofractionation, despite being radiobiologically superior to melanoma cells, seems to be limited in the head and neck region by proximity of important normal tissues sensitive to higher fraction doses [20].

Considering the low rate of spread to the CLN, we advocate elective neck dissection only for selected patients with a high local disease burden or with a primary tumor located in or extending to the oral cavity or oropharynx. In the present series, of 12/61 patients presenting with clinically positive CLN, six had neck dissection (pN+ disease confirmed in all cases) and two received definitive neck irradiation. Besides these, two patients with pT4a tumors and without clinically positive CLN were treated with neck dissection as well, both being classified as pN0. Overall, of 48 patients treated with curative intent 17 % later relapsed in the CLN. However, this figure must be interpreted with caution, because as much as 48 % of the patients had only clinical examination of the neck performed during primary diagnostics. In the literature, there has been much controversy over elective treatment of CLN. The latter is supported by some authors [15, 21] while others oppose elective neck dissection in MMHN because of low rates of positive CNL and regional recurrences, proposing only a wait-and-see policy [22]. An appealing approach to clarify the need for elective neck dissection in MMHN patients is sentinel lymph node biopsy, which is an already well established diagnostic tool in cutaneous malignant melanoma. As only case reports on this subject have been published so far [23], further research is needed to determine its value in diagnostic algorithms for MMHN.

A combination of immunotherapy and chemotherapy (biochemotherapy) has previously showed considerable response rates and favorable effect on progression-free interval but with no impact on OS [24]. Our data from multivariate analysis, controlled for age, PS WHO and treatment modality, confirm the lack of effect of systemic treatment on survival. However, new targeted drugs are constantly being developed and their effect on disease course seems more promising. Taking into account results from cutaneous melanoma studies, the minority of MMHN patients with driver mutations of oncogenes could benefit from KIT and BRAF inhibitors [25, 26]. The aggressive behavior of MMHN presents a great challenge to clinicians. Even in the era of advanced diagnostic and treatment options prognosis remains dismal and little progress in the outcome of these patients was observed. In 1998 Chang et al. reported on 212 MMHNs, diagnosed between 1985 and 1989, with a 5-year overall survival rate of 31.7 % which is not notably worse than most favorable results reported in the more recent series (Table 5) [3, 13, 19, 27–37]. These results suggest that at least a temporary plateau has been reached concerning survival. The herein presented results of multivariate analyses of survival possibly reflect this finding, as only performance status was found to be an independent prognosticator of OS. More advanced surgical and irradiation techniques used in more recent series probably resulted in improved treatment-related toxicity profile, contributing to less detrimental effect on post-treatment quality of life [32]. Nine patients in the present series who survived more than 5 years (3/9 lived more than 10 years) after diagnosing MMHN confirm the potential of achieving long survival times in MMHN. The retrospective nature of the present and previously published MMHN series is inherent to rare cancer studies. This results in notable treatment selection bias which in turn hampers analyses of prognostic factors. Furthermore, the majority of MMHN series are single- or multi-institutional, leading to possible referral bias. In contrast, the present series is a population-based study and as such offers to fill this potential data gap. In addition, we performed controlled, multivariate analyses of prognostic factors, further contributing to the quality of the presented results.

**Table 5:** Mucosal melanoma of the head and neck: a review of the recent literature series (with ≥50 patients & utilizing radiotherapy)

Author	Setting, country	Period of diagnosis	No. of patients	Age	Primary tumor site (%)	TNM stage (%)	Treatment modality (%)	OS (at 5 years, %)	DSS, (at 5 years, %)
				Median/Mean* (range)					
Gal et al., 2011 [29]	Population-based (26 %), USA	2000–2007	304	71.2*	SN, all	III, 32.2	S, 43.1	24.2	NR
						IVA, 25.3	SRT, 38.5		
						IVB, 11.2	RT, 7.6		
						IVC, 12.2	None/Unknown, 10.9		
Jangard et al., 2013 [13]	Population-based (96 %), Sweden	1960–2000	186	72 (31–93)	SN, all	I, 83.9	S, 53.1	NR	20.4
						II, 2.2	SRT, 32		
						III, 4.3,	SRTSTh, 2.7		
						Unknown, 9.7	Palliative, 12.2		
Benlyazid et al., 2010 [30]	Multi-institutional, France	1980–2008	160	67.0 (30–93)	SN, 90.6	I, 96.3	S, 51.3	37.5	NR
					OC, 7.5	II, 3.8	SRT, 48.8		
					Other, 1.9				
Lund et al., 2012 [27]	Single institution, UK	1963–2010	115	65.9* (15–91)	SN, all	I, 87.8	S, 55.7	28	NR
						II, 8.7 Unknown, 3.5	SRT, 44.3		
Shiga et al., 2012 [31]	Multi-institutional, Japan	1998–2007	94	68.4* (37–96)	SN, 78.7	I, 16.0	S, 9.6	S + STh/RT, 38.6	NR
					OC-OP, 15.6	II, 28.7	RT, 9.6	RT+/−STh, 29.9	
					Unknown primary, 3.2 External ear, 2.1	III, 9.6	STh, 7.4		
						IVA, 31.9	S + STh/RT, 52.1		
						IVB, 3.2	RT+/−STh, 19.1		
						IVC, 10.6	None, 2.1		
Meng et al., 2014 [32]	Single institution, China	2000–2010	69	65.9* (28–89)	SN, all	III, 53.6	S, 39.1	S, 31.6	NR
						IVA, 39.1	SRT, 34.8	SRT, 55	
						IVB, 7.2	SRTSTh, 26.1	SRTSTh, 32.1	
Sun et al., 2014 [33]	Single institution, China	1976–2005	68	55 (2–79)	SN, all	III, 52.9	S, 27.7	29.7	NR
						IVA, 35.3	RT, 6.2		
						IVB, 5.9	STh, 12.3		
						IVC, 5.9	SRT, 20.0		
							S + STh, 29.2		
							RT + STh, 3.1		
							SRTSTh, 1.5		
Douglas et al., 2010 [34]	Single institution, UK	1965–2001	68	63 (29–86)	SN, 65	I, 80	S, 27	22	32
					OC, 19	II, 19	RT, 46		
					Other, 16	III, 1	SRT, 7		
							Palliative, 20		
Demizu et al., 2014 [35]	Single institution, Japan	2003–2011	62	70.5 (33–89)	SN, 90.3	T1, 27	RT, all	At 1 year, 93	NR
					OC, 9.7	T2, 31	- protons, 53.2	At 2 years, 61	
						T3, 31	- carbon ion, 46.8		
						T4, 11	Prior treatment:		
							S, 11.3		
							STh, 8.1		
							S + STh, 3.2		
Moreno et al., 2010 [19]	Single institution, USA	1993–2004	58	63.4 (38–93)	SN, all	I, 87.9	S, 43.1	38.7	18.4
						II, 6.9	SRT, 53.4		
						III, 5.2	RT, 3.4		
Shuman et al., 2011 [28]	Single institution, USA	1992–2009	52	66*	OC, 31	I, 25.0	S, 69.2	38	22
					SN, 69	II, 34.6	SRT, 19.2		
						III, 11.5	Palliative RT, 9.6		
						IV, 28.8			
Sun et al., 2012 [36]	Single institution, China	1976–2005	51	55 (31–75)	OC, all	III, 23.5	S, 76.5	20.7	NR
						IVA, 34	SRT, 3.9		
						IVB, 4	STh, 11.8		
						IVC, 1	None, 7.8		
Koivunen et al., 2012 [37]	Multi-institutional, Finland	1990–2004	50	70* (46–93)	SN, all	III, 36	S, 66.0	27	48
						IVA, 42	SRT, 14.0		
						IVB, 20	RT, 14.0		
						IVC, 2			
Current series, 2016	Population-based, Slovenia	1986–2013	61	75.5 (25–91)	SN, 72	III, 34	Curative intent	Whole cohort, 18 Curative intent, 24	Curative intent, 25
					OC-OP, 26	IVA, 49	(*N* = 48):		
					Middle ear, 1.6	IVB, 12	S, 44		
						IVC, 4.9	SRT, 33		
							RT, 23		

*OS* Overall survival, *SN* Sinonasal tract, *S* Surgery, *SRT* Surgery and postoperative radiotherapy, *RT* Radiotherapy, *NR* Not reported, *SRTSTh* Surgery with postoperative radiotherapy and systemic therapy, *OC* Oral cavity, *OC-OP* Oral cavity and oropharynx, *STh* Systemic therapy

*Mean age

## Conclusions

MMHN is a rare cancer with a stable incidence of far below two cases per million. For this reason, unbiased data on the optimal treatment is lacking. However, MMHN's propensity to recur even after radical surgical treatment is well known and this dictates the need for aggressive adjuvant treatment. The results presented herein support the use of high-dose PORT in selected patients in whom LRC is deemed worth the side effects. The roles of elective treatment of the CLN and of systemic treatment remain to be elucidated.

## Abbreviations

CI: Confidence interval
CLN: Cervical lymph nodes
CT: Computer tomography
DMFS: Distant metastasis-free survival
EQD_2_: Equivalent doses in 2-Gy fractions
HR: Hazard ratio
LC: Local control
LRC: Locoregional control
MMHN: Mucosal melanoma of the head and neck
MRI: Magnetic resonance imaging
N: Number of patients
NA: Not applicable
NI: Not included
NR: Not reported
OC-OP: Oral cavity and oropharynx
OS: Overall survival
PET: Positron emission tomography
PORT: Postoperative radiotherapy
RC: Regional control
SN: Sinonasal tract
SRTSTh: Surgery with postoperative radiotherapy and systemic therapy
STh: Systemic therapy
US: Ultrasonography
WHO PS: World Health Organization performance status
